# Supplementary material for: The interactome of intact mitochondria by cross-linking mass spectrometry provides evidence for coexisting respiratory supercomplexes
Source: Mol Cell Proteomics. 2017 Dec 8;17(2):216–32. doi: 10.1074/mcp.RA117.000470 (PMC5795388; doi:10.1074/mcp.RA117.000470)
Supplement: Supplemental Data [file supp_RA117.000470_133922_0_supp_23978_fzffwf.zip › spectra_annotation/mito_spectra_annotation/377-1-2-1-5-1.pdf]

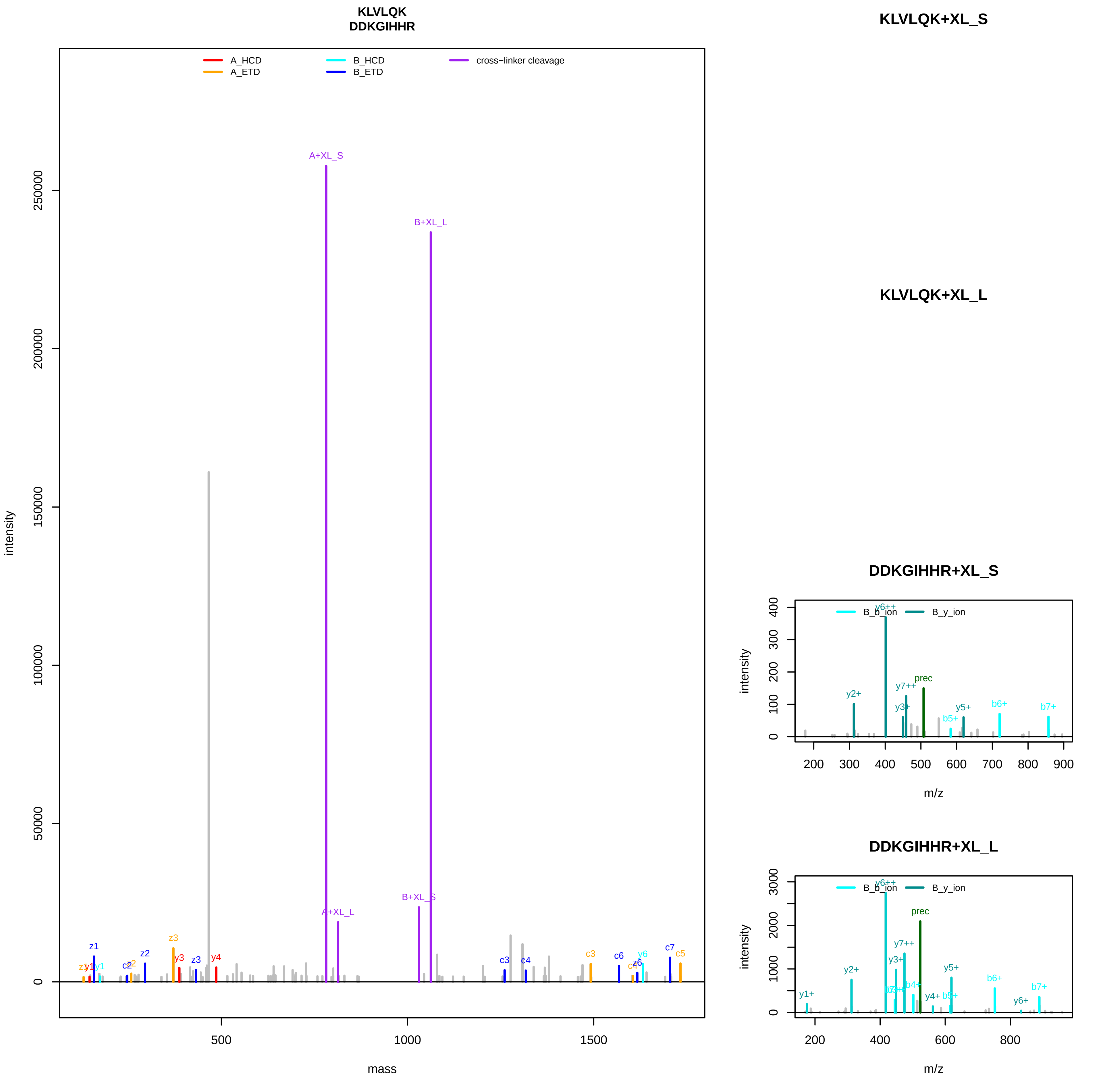
KLVLQK
DDKGIHHR
A_HCD
A_ETD
B_HCD
B_ETD
cross−linker cleavage
A+XL_S
B+XL_L
A+XL_L
B+XL_S
intensity
mass
0
50000
100000
150000
200000
250000
500
1000
1500
z1
y1
c2
z2
z3
y3
y4
c3
c4
c6
z6
y6
c7
c5
KLVLQK+XL_S
KLVLQK+XL_L
DDKGIHHR+XL_S
B_b_ion
B_y_ion
y6++
y2+
y7++
y3+
prec
y5+
b5+
b6+
b7+
m/z
0
100
200
300
400
200
300
400
500
600
700
800
900
DDKGIHHR+XL_L
y1+
y4+
y6+
b4+
0
1000
2000
3000
200
400
600
800
